# Conditionally active antibody‒drug conjugates

**DOI:** 10.1016/j.apsb.2026.08.023

**Published:** 2026-08-31

**Authors:** Cuiyu Guo, Yaqin He, Ying Lu, Qilin Dun, Jinliang Yang, Yuxi Wang

**Affiliations:** a Department of Pulmonary and Critical Care Medicine, Targeted Tracer Research and Development Laboratory, Institute of Respiratory Health; State Key Laboratory of Respiratory Health and Multimorbidity; West China Hospital, Sichuan University, Chengdu 610041, China; b State Key Laboratory of Biotherapy and Cancer Center, West China Hospital, West China Medical School, Sichuan University, Chengdu 610041, China; c Frontiers Medical Center, Tianfu Jincheng Laboratory, Chengdu 610212, China

**Keywords:** Conditionally active ADCs, Prodrug, Tumor-targeted therapy, Tumor microenvironment, Drug development, Next-generation ADCs, Masking-activation, Probody platform

## Abstract

Conditionally active antibody‒drug conjugates (ADCs) can be achieved through antibody engineering and/or linker-payload design to achieve context-dependent activation, addressing the limitations of conventional ADCs such as premature payload release, inefficient tumor penetration, and on-target, off-tumor toxicities. Antibody surfaces can be modified with steric shields, site-specific conjugation, or reversible masking groups to further limit off-target binding. Linker-payloads are masked in circulation through prodrug strategies, such as multi-step or logic-gated linkers, releasable caps, or bioorthogonal chemistries. These strategies confine cytotoxic drugs to the tumor microenvironment (TME), enhancing intratumoral drug concentration, expanding the therapeutic window, and reducing systemic toxicity. Early clinical studies of conditionally active ADCs targeting ROR2, AXL, and CD71 have demonstrated promising safety and efficacy. This review discusses the underlying mechanisms, antibody engineering and linker-payload design strategies, and clinical progress of conditionally active ADCs, and considers future avenues to improve tumor selectivity and therapeutic performance.

## Graphical abstract

Conditionally active ADCs incorporate controllable “masking-activation” mechanisms at the antibody, linker, and payload levels, enabling the ADC to remain inert during systemic circulation while selectively activated in the tumor microenvironment, thereby achieving precise drug delivery and reduced off-target toxicity.

**Figure:**
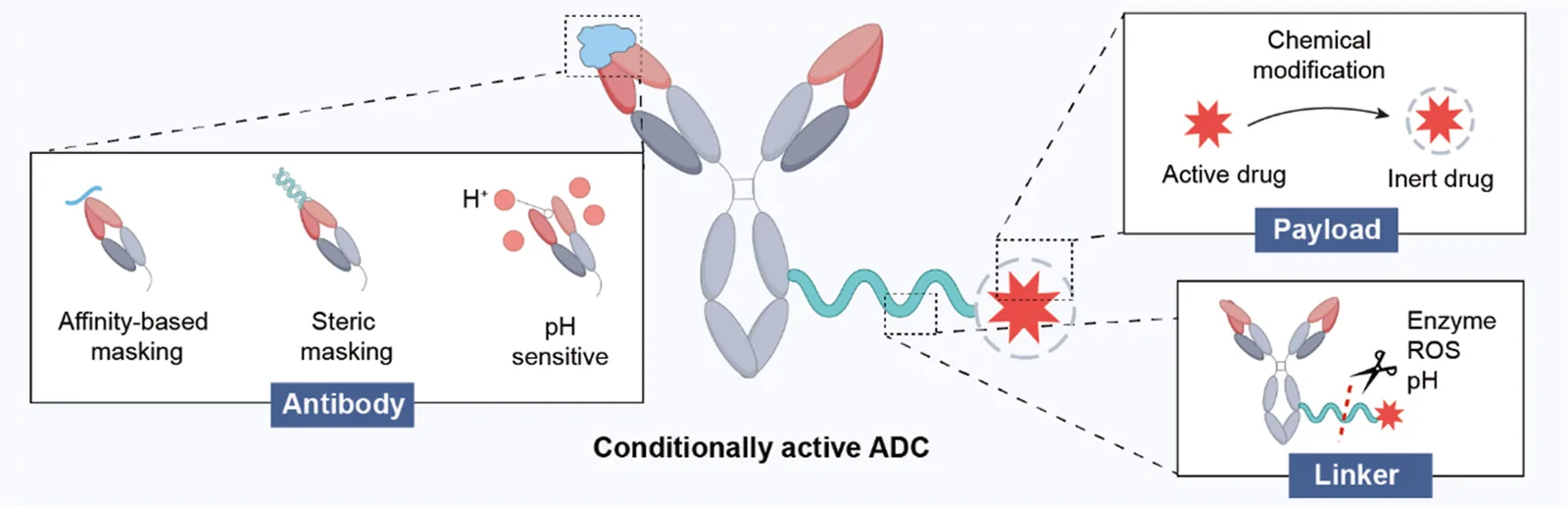

## 1 Introduction

Over the past decade, the rapid advancement of antibody‒drug conjugates (ADCs) has reshaped the therapeutic landscape of cancer^1^. ADCs harness the specificity of antibodies to deliver potent cytotoxic drugs in a targeted manner, functioning as a form of “biologically guided chemotherapy” (Fig. 1A). Despite structural heterogeneity, ADCs generally share a common mechanism of action: antigen binding, cellular internalization, lysosomal processing and payload release, culminating in tumor cell death (Fig. 1B)^2^^,^^3^. By November 2025, over twenty ADCs have been approved globally, covering a range of solid and hematologic malignancies including breast cancer, lymphoma, gastric cancer, lung cancer, and urothelial carcinoma^4^. However, ADCs face several fundamental challenges in clinical application. Premature linker cleavage in circulation, nonspecific payload distribution, and plasma protein binding also compromise delivery specificity, resulting in less than 1% of administered ADCs effectively reaching tumor tissue^5^. Heterogeneous expression of target antigens in normal tissues can lead to unavoidable on-target, off-tumor toxicities, thereby constraining dose escalation, narrowing the therapeutic window, and, in some cases, resulting in drug withdrawal (Fig. 1C)^6^.

**Figure 1:**
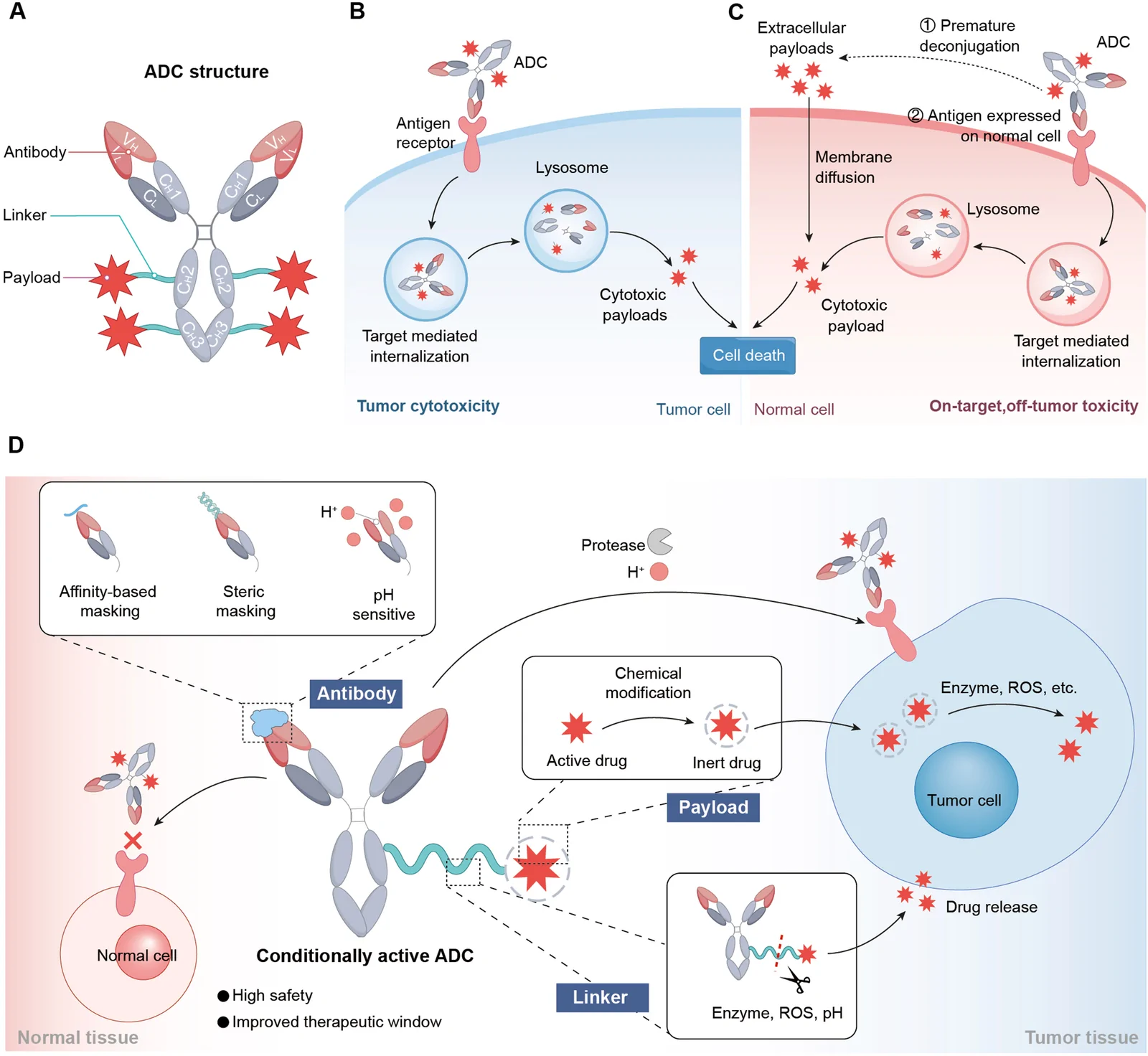
Mechanisms of action of ADCs, sources of clinical toxicity, and design principles of conditional activation. (A) ADC is composed of a specific antibody, a cytotoxic payload, and a linker that connects them. (B) Mechanism of action for conventional ADCs: after binding to the antigen receptor, ADCs are internalized into tumor cells, where the payload is released within lysosomes to induce cell death. (C) The therapeutic index of ADCs is limited by several factors, including on-target, off-tumor toxicity due to antigen expression in normal tissues, non-specific cell killing caused by linker instability, and payload membrane diffusion. (D) Design framework for conditionally active ADCs: by introducing masking agents or chemical switches responsive to TME cues (*e.g.,* enzyme, ROS, pH) at the antibody, linker, or payload, ADCs remain inert in normal tissues and are selectively activated within tumor regions. This approach expands the therapeutic window and reduces systemic toxicity.

Recent insights into the tumor microenvironment (TME) indicate that uncontrolled proliferation and aberrant metabolism of malignant cells drive the TME far from normal tissue homeostasis. The alterations include upregulated protease activity, chronic hypoxia, acidic pH, increased reducing metabolites, and abundant extracellular nucleotides and adenosine triphosphate release^7^^,^^8^. These signals provide endogenous cues exploited to design conditionally active ADCs, which remain inert in normal tissues yet become selectively activated within the TME, marking a paradigm shift from conventional passive targeting to stimuli-responsive delivery. Several conditionally active ADCs are currently in development, with a subset entering clinical evaluation^9^, ^10^, ^11^. Related strategies primarily focus on three core mechanisms: (i) engineering masked antibodies with reduced affinity in normal tissues, selectively reactivated by tumor-enriched proteases; (ii) incorporating reversible, environment-responsive modules, such as pH-sensitive binding proteins, to achieve selective engagement within the acidic TME; (iii) introducing chemical switches into the linker and/or payload to confine cytotoxic release to tumor-restricted conditions (Fig. 1D). Preliminary studies indicate that conditionally active ADCs can potentially enhance intratumoral drug concentration, expand the therapeutic window, and reduce systemic toxicity; however, they remain in early development, and their activation thresholds, response kinetics, *in vivo* predictability and safety still require further investigation.

Based on the above, this review systematically summarizes the design frameworks for conditionally active ADCs, including antibody-based activation strategies, chemically responsive linker-payload modules, and recent advances in multi-trigger activation, programmed release, and exogenous stimuli-responsive mechanisms. We also discuss current technical bottlenecks and propose potential evolutionary directions for next-generation ADCs, providing theoretical foundations and design guidance for safer, more precise, and controllable tumor-targeted therapies.

## 2 Design principles for conditionally active ADCs

Biochemically distinct features of the TME present intrinsic opportunities for designing conditionally active ADCs^8^. Proteases such as matrix metalloproteinases (MMPs), urokinase-type plasminogen activator (uPA), membrane-type serine protease 1, and legumain are broadly upregulated during tumor invasion and metastasis but remain tightly regulated in normal tissues, enabling high tumor specificity^12^. Tumors preferentially rely on aerobic glycolysis, resulting in lactate and proton accumulation and a decrease in extracellular pH to ∼5.8‒6.5, whereas normal tissues maintain a near-neutral pH of ∼7.4^13^. Malignant cells display persistent redox imbalance, reflecting sustained reactive oxygen species (ROS)-generating pathways and chronic oxidative stress, while normal cells preserve redox homeostasis *via* tightly controlled antioxidant systems^14^^,^^15^. The following sections outline how different ADC components can be conditionally activated by these endogenous tumor-associated cues, as well as examples of ADCs activated by exogenous stimuli.

### 2.1 Antibodies-based conditionally active ADCs

#### 2.1.1 Protease-triggered masked antibodies

Engineering of the antibody moiety is integral to ADC design, ensuring optimal targeting, internalization, and conjugation performance. Masked antibodies are engineered to remain in a “silent” state in normal tissues, minimizing off-target toxicity, by attaching a cleavable masking moiety to the N-terminus of the light or heavy chain, which sterically occludes the antigen-binding site^16^^,^^17^. When exposed to proteases or other stimuli abundant in the TME, the mask is removed or deactivated, reinstating antigen-binding function. Existing designs broadly fall into two categories: affinity-based masking and steric masking.

Affinity-based masking typically relies on domains that specifically bind to the antibody paratope, such as anti-idiotypic scFv, nanobodies, or peptide mimotope^18^, ^19^, ^20^. A prime example is CytomX's Probody platform, in which an affinity peptide, flexible linker, and protease-cleavable substrate are fused to the light chain, keeping the antibody in a masked state in circulation while allowing selective activation by MMPs/uPA (Fig. 2)^18^^,^^19^. Several tumor antigens that have historically proven difficult or unsafe to target with traditional ADCs, such as CD71, CD166, and EpCAM, are now being revisited using conditionally activated ADC approaches, with early clinical studies demonstrating narrow toxicity profiles and measurable tumor regression^20^, ^21^, ^22^. The masking peptide's affinity must be carefully tuned to ensure complete restoration of antigen binding following protease-mediated unmasking. Kolmar et al.^20^ developed a generic strategy to enable reliable and rapid access to affinity-based masking units from a chicken-immunized library, and achieved effective blockade of the interaction between the 6G11 antibody and its receptor Fc*γ*RIIB by fusing an anti-idiotypic scFv to the N-terminus of the antibody light chain (S2B-6G11). In this study, the masking units obtained through screening still exhibited residual binding after proteolytic cleavage; therefore, to achieve effective off-tumor masking while restoring antibody function within the TME, additional rational design was applied to tune the masking domain into an optimal “affinity window”^20^. This masked antibody is expected to mitigate potential clinical side effects arising from unintended engagement of Fc*γ*RIIB by 6G11, which could otherwise interfere with multiple physiological processes.

**Figure 2:**
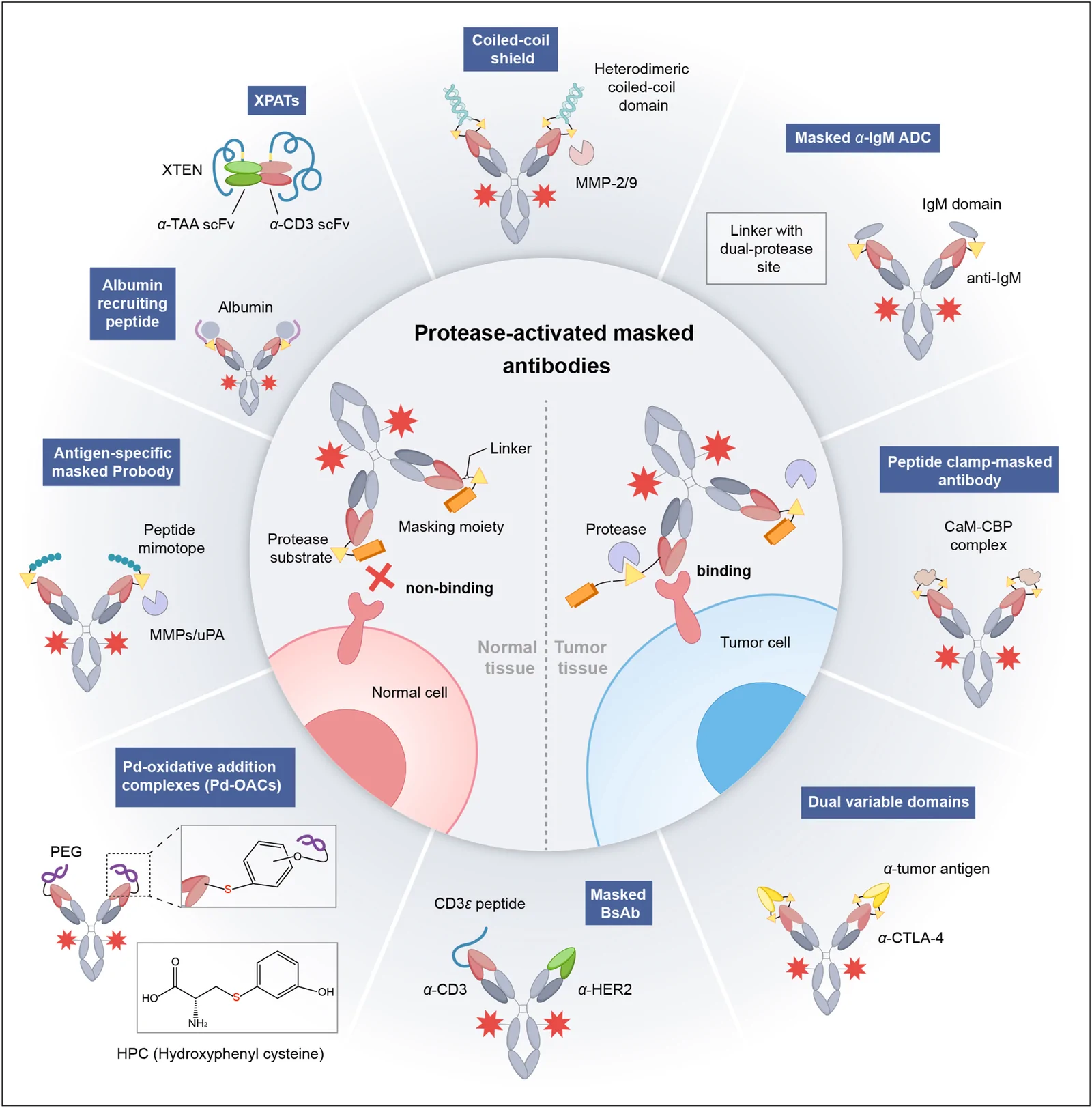
Protease-triggered masked antibody strategies. In the central schematic, the masked antibody does not bind to receptors on the surface of normal cells in healthy tissues, but regains antigen recognition within tumor tissue upon protease-triggered activation. Surrounding examples illustrate diverse approaches: (from the left, in a clockwise order) incorporation of a protease-cleavable mimotope at the N-terminus of the antibody to enable conditional unmasking; steric shielding using macromolecular polymers, intrinsically disordered peptides (*e.g.*, XTEN), or albumin recruitment; steric masking *via* coiled-coil motifs, IgM domain and peptide clamps that dissociate controllably. Some designs further integrate dual-variable domain or bispecific architectures. And site-specific chemical installation of cleavable non-natural amino acids or polymeric shields at the antigen-binding interface (Pd-OACs).

Despite its advantages, affinity-based masking is highly antigen-specific, complicating modular transfer between different antibody frameworks and potentially challenging PK prediction in multispecific constructs. In contrast, steric masking operates independently of specific paratope-mask interactions, providing broad applicability and facilitating modular transfer across diverse antibodies. Including extended polypeptide chains (*e.g.,* XTEN), albumin-binding domains, and engineered folded domains from antibody constant regions^23^, ^24^, ^25^, ^26^. The volume and conformation of masking moieties critically influence blocking efficiency^27^. Seagen employed heterodimeric coiled-coil structures to mask antibody complementarity determining region (CDRs), significantly reducing affinity in healthy tissues while restoring activity in MMP-2/9-enriched TME (Fig. 2)^28^. The resulting ADC exhibited an extended half-life and enhanced antitumor efficacy compared with the unmodified ADC in preclinical models. Further expanding the design space, Kolmar et al.^26^^,^^29^ employed human IgM CH2 domains as cleavable masks regulated by dual protease substrates (MMP site and MatA site), as well as compact calmodulin-binding peptide (CaM-CBP) complexes to occlude the paratope. Masked CH2-aIgM reduces binding to IgM^+^ cells by 61-fold (SUP-B8 cell) and 102-fold (Ramos cell) and completely blocks ADC-mediated cytotoxicity. After protease cleavage, the binding affinity is restored to 1.5‒2.6 nmol/L, and maximal cell killing is comparable to the unmasked antibody, thereby improving target specificity and safety. The human CaM-CBP peptide clamp efficiently masks antibodies, reducing on-cell binding by 140-fold (for trastuzumab) and 257-fold (for cetuximab), while MMP-9 cleavage restores binding and effector function to unmodified levels. ADCs constructed using these steric masking strategies allow selective elimination of tumor or malignant cell populations while sparing normal tissues.

Multifunctional shielding structures can simultaneously achieve targeted positioning and extended half-life. For instance, conditionally active dual-variable domain (DVD) bispecific antibodies fuse a second pair of variable regions to the N-terminal end of the anti-CTLA-4 chain (Fig. 2)^30^. Unshielding occurs *via* a short linker peptide cleavable by membrane-type serine proteases overexpressed in the TME, thereby preserving antitumor effects while minimizing immune activation in non-tumor tissues. Anti-CD79b DVD IgG1 ADCs exhibit highly potent, antigen-specific cytotoxicity, retain full activity after 72 h in human or mouse plasma, and show minimal toxicity toward CD79b-negative cells, highlighting their superior stability and selective payload delivery compared with traditional ADCs^31^. More sophisticated masking agents can integrate multifunctional engineering, such as cleavable motifs for bispecific antibodies, enabling precise temporal and spatial control over binding to both targets. This approach mitigates cytokine release syndrome, a common complication in T cell redirection therapies^32^.

Most existing masked antibodies rely on recombinant expression, introducing masking and cleavable modules at the N-terminus to generate fusion proteins. This strategy limits masking groups to the 20 canonical amino acids and necessitates oligomeric forms compatible with recombinant expression. Recent chemical approaches provide greater flexibility. For instance, palladium-mediated cysteine arylation installs *ortho*-hydroxyphenyl groups onto cysteine residues *via* a Pd-catalyzed oxidative addition. Protease-responsive sequences or PEG-based masks can then be conjugated to form Pd-oxidative addition complexes (Pd-OACs) that are efficiently removed by tumor-associated proteases, ultimately revealing an *S*-aryl cysteine-hydroxyphenyl cysteine (HPC) motif (Fig. 2)^33^. This chemical strategy enables the site-specific construction of antibody prodrugs within CDRs, circumventing the traditional restriction of masking groups to the N-terminus while providing precise control over the masking site.

#### 2.1.2 pH-dependent antibodies

Although protease-activated masked strategies are elegant, their activation is typically irreversible. Once unmasked, antibodies may diffuse beyond the tumor into normal tissues, potentially causing off-target binding and toxicity. To address this issue, specific signals from the acidic TME have been sought to achieve reversible activation. One such approach employs engineered pH-responsive variants of protein M from Mycoplasma genitalium to reversibly mask trastuzumab. At physiological pH, the variant binds the antibody light-chain Fv with high affinity, effectively silencing antigen recognition. In the acidic TME (pH∼6.0‒6.5), this interaction is weakened, gradually restoring HER2 binding^34^. Beyond masking-based strategies, direct engineering of low pH-selective binding antibodies offers an alternative means to enhance ADC tumor penetration. A B7-H3-targeting acidic-selective ADC (AR153) displays minimal cytotoxicity under neutral conditions but achieves potency comparable to conventional ADCs at acidic pH (6.0). In B7-H3-positive cell line-derived xenograft models, both constructs elicit pronounced antitumor responses; however, AR153 demonstrates superior intratumoral penetration, enabling selective tumor targeting while sparing normal tissues^35^.

The fundamental molecular mechanism underlying pH-sensitive antibody recognition is the protonation dynamics of histidine residues. Johnston et al.^36^ revealed that the immune checkpoint receptor V-domain immunoglobulin suppressor of T cell activation (VISTA) engages P-selectin glycoprotein ligand-1 (PSGL-1) *via* a histidine-rich binding interface, resulting in strict pH dependence and functional activity confined to acidic environments. Leveraging this property, pH-selective antibodies have been designed to specifically recognize the protonated, active form of VISTA, selectively inhibiting VISTA–PSGL-1 interactions under acidic conditions while limiting systemic engagement, target-mediated drug disposition, and cytokine release syndrome. More broadly, histidine engineering within antibody CDRs offers a generalizable approach to confer acidic pH selectivity through structure-guided design. Even introducing a single histidine residue at the interface significantly enhances pH-dependent binding. *De novo* structure-guided design integrating histidine into the HER2 antibody binding interface reduced binding affinity by approximately 10-fold at pH 7.3 while maintaining low nanomolar affinity across the pH 6.0‒6.8 range^37^.

BioAtla's Protein-associated Chemical Switches (PaCS) screening platform enables the identification of pH-sensitive antibodies even in the absence of histidine-rich target epitopes, thereby achieving highly selective activation without the need for explicit amino acid engineering (Fig. 3A). This approach constructs a library of CDR variant proteins and screens them in enzyme-linked immunosorbent assay (ELISA) under simulated physiological ionic conditions, including sodium chloride, sodium bicarbonate, sodium sulfide, and lactic acid. Based on PaCS screening, BioAtla has developed pH-dependent ADCs targeting AXL and ROR2, both currently in Phase II clinical trials^38^, ^39^, ^40^. Additionally, Chang et al.^41^ developed anti-CTLA-4 antibodies using PaCS technology, exhibiting an EC_50_ at pH 7.4 that was only half that at pH 6.0. Low pH-selective antibodies were also obtained for targets including EpCAM, HER2, Nectin4, CD73, and CD3. Mechanistically, pH responsiveness is generally conferred by substituting polar or nonpolar residues with charged amino acids, such as aspartate, glutamate, or histidine, within the antigen-binding interface.

**Figure 3:**
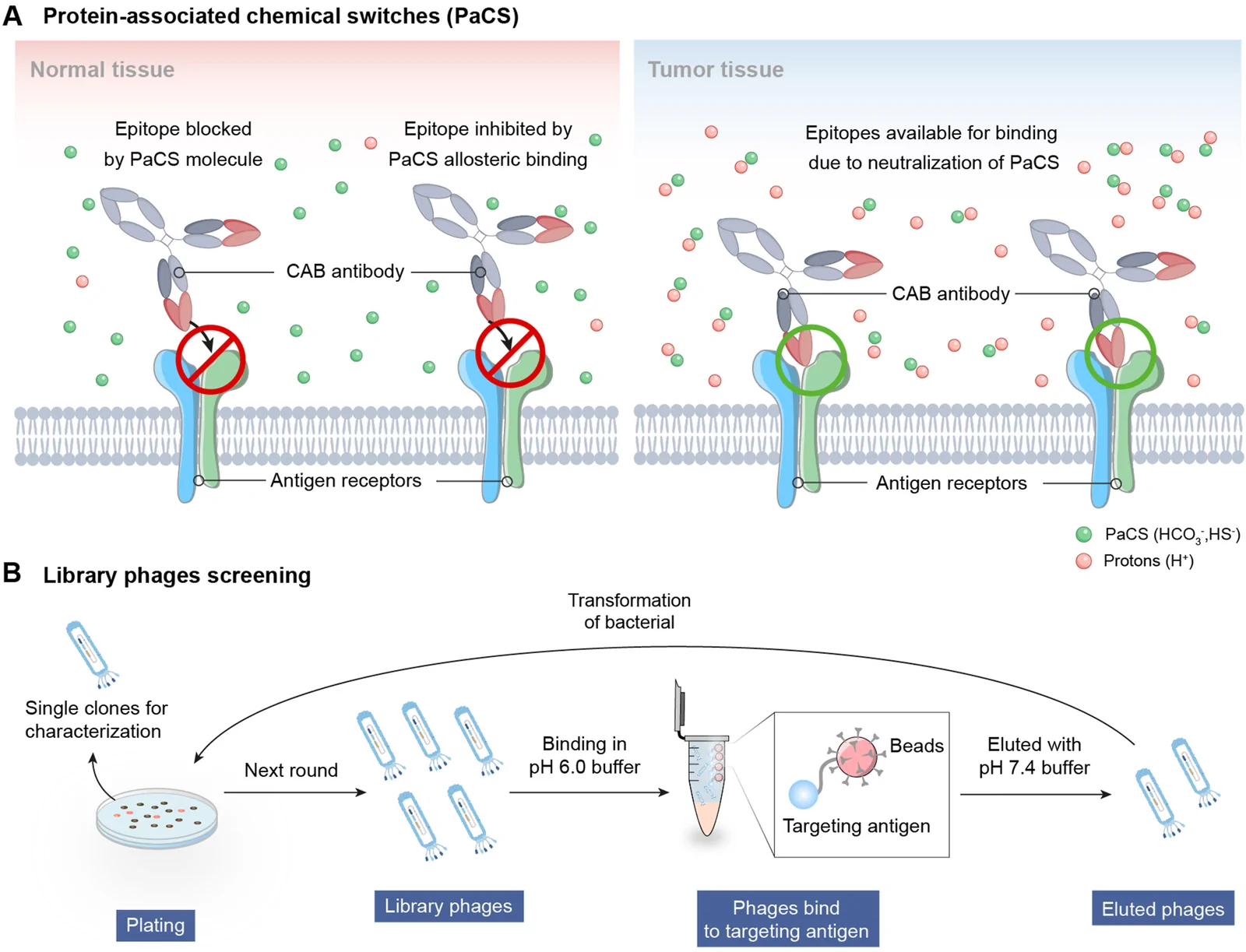
pH-dependent antibody strategies and screening process. (A) Protein-associated chemical switch (PaCS) mechanism. Endogenous physiological molecules, such as bicarbonate and hydrogen sulfide, typically carry negative charges under normal conditions and interact with positively charged regions on the antibody surface. In the acidic TME, these molecules become neutralized and dissociate from the protein, selectively relieving inhibition and enabling conditionally active biologics (CAB) antibodies to bind their targets and mediate tumor cell killing. (B) Phage display-based low pH-selective antibody screening workflow. Antibody libraries bind to target antigens under acidic conditions and are eluted at physiological pH to enrich antibody clones exhibiting pH-selective binding characteristics for subsequent characterization and optimization.

Strategies for further optimizing pH-dependent antibodies include structure-guided library design and phage display screening to enhance the binding affinity ratio between acidic pH and physiological pH (*i.e.*, selectivity) while reducing binding to normal tissues (Fig. 3B)^42^^,^^43^. The engineered pH-sensitive anti-EGFR antibody G532 exhibits a 13-fold increase in EGFR affinity within the TME compared to physiological pH (*K*_d_ = 294 nmol/L *vs.* 3.9 μmol/L), significantly enhancing tumor penetration and antitumor activity^44^. Introducing H-D/E interactions effectively modulates pH dependence at the antibody-antigen interface, while tyrosine derivatives (*e.g.*, 3-nitro-l-tyrosine, 3-cyano-l-tyrosine, 3,5-halogenated-l-tyrosine) can act as pH sensors within the pH 6.0‒7.4 range, further optimizing selective binding^45^.

Because the number and spatial arrangement of pH-sensitive residues affect activity, tailored engineering strategies continue to depend on integrated molecular modeling and *in vitro* evolution techniques. Recent advancements in artificial intelligence and machine learning have substantially enhanced design efficiency. Sequence-directed approaches like SIpHAB can predict histidine mutations conferring acid or physiological pH sensitivity to antibodies^46^. Meanwhile, deep learning (DL)-based protein design can now *de novo* generate stable pH-sensitive binders^47^. By guiding histidine design through hydrogen bonding and electrostatic repulsion mechanisms, these methods achieve stronger binding at low pH. Modulating the p*K*_a_ of histidine residues by altering surrounding residues enables optimal activation at pH ∼6.5, thereby improving therapeutic window and TME selectivity.

### 2.2 Conditionally active linker and payload design

Traditional ADC designs often rely on single-trigger linker, such as enzyme-cleavable peptides (*e.g.*, VC and GGFG), glutathione-sensitive disulfide bonds, and pH-sensitive chemical bonds (acetal, ortho-ether, imine, hydrazone, *β*-lactam bonds, etc.). Despite conferring partial tumor selectivity, these linkers are still susceptible to off-target cleavage in circulation or normal tissues, resulting in premature drug release and limit therapeutic index^48^^,^^49^. Integrating TME-responsive mechanisms, sensitive to specific enzymes, acidic pH, redox states, or their combinations, into linker-payload design, and evolving from single-to dual- or programmable-response systems, offers enhanced spatiotemporal control over drug delivery (Fig. 4A). For example, in linker-payload design, hydrophobicity frequently undermines protein labeling efficiency and fosters both aggregation and off-target toxicity^50^^,^^51^. Two-step cleavage linker strategies, such as benzyl *α*-ammonium carbamates (BACs), achieve dual-step cleavage through a tandem 1.6–1.2 elimination reaction (Fig. 4B, **1**)^52^. This design shields the hydrophobic payload during circulation, enhancing *in vivo* stability, and can be combined with protease cleavage or terminal trigger groups. Similar strategies include glycopeptide linkers (**2**) that introduce glucuronide or galactosyl groups *via* serine, enabling two-step enzymatic cleavage. This approach ensures plasma stability while achieving lysosomal-specific activation^53^^,^^54^.

**Figure 4:**
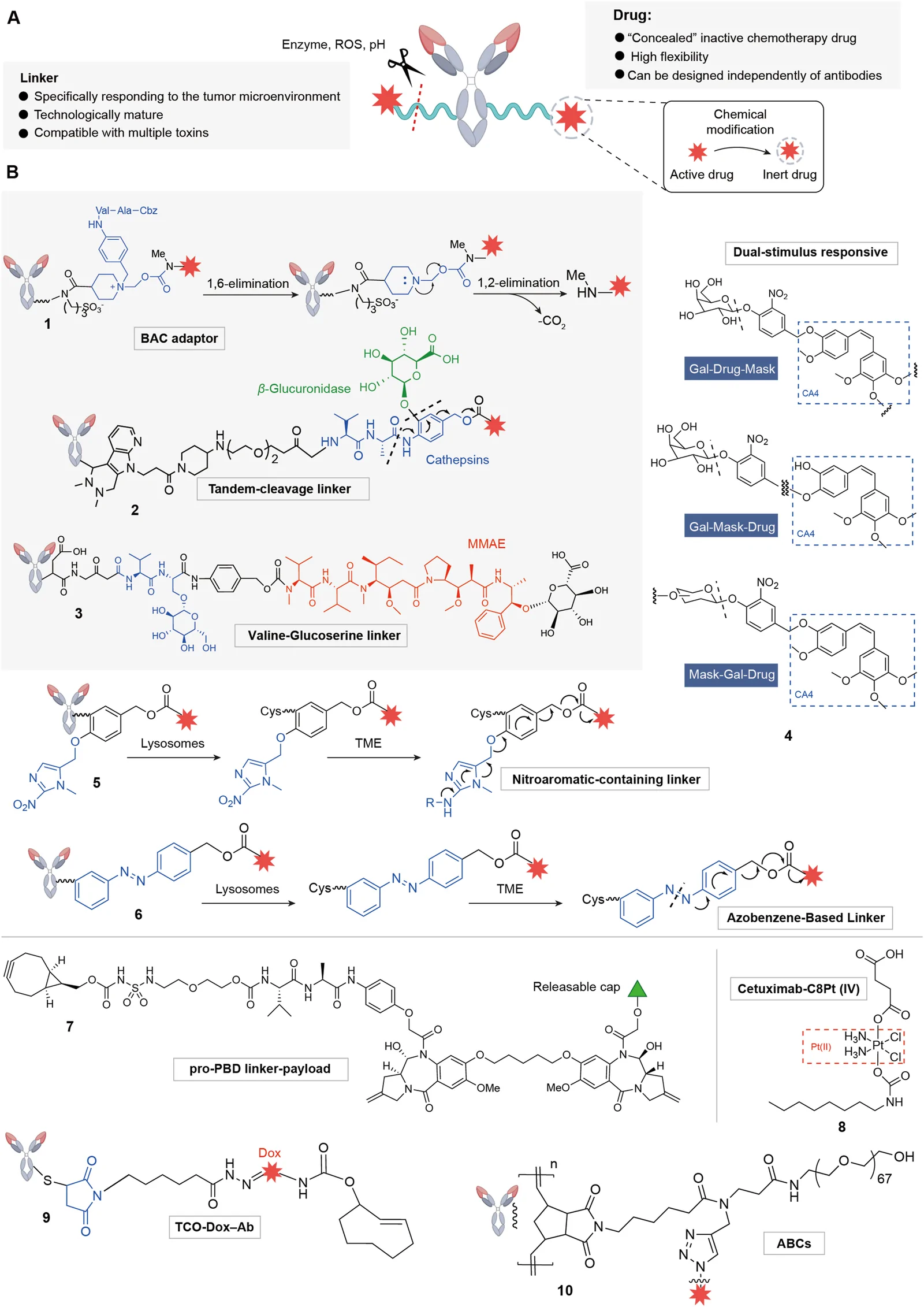
Design strategies for linkers and payloads in conditionally active ADCs. (A) By achieving conditional cleavage and drug release in response to characteristic TME stimuli, the linker-payload module offers enhanced spatiotemporal control over drug delivery. (B) Representative examples of conditionally active linker-payload designs: dual-step benzyl *α*-ammonium carbamates (BACs) linkers (**1**); tandem cleavage linkers dependent on *β*-glucuronidase or cathepsin (**2**); lysosome-specific linkers based on glycopeptides or amino acid derivatives (**3**); glycosylated prodrug systems with dual-stimulus response logic (**4**); hypoxia-activated nitroaromatic or azobenzene linkers for ultra-potent payloads (**5,6**); pro-pyrrolobenzodiazepine (Pro-PBD) designs for reduced toxicity (**7**); Pt(IV) metal prodrugs converted to Pt(II) in tumors (**8**); bioorthogonal and TME-responsive doxorubicin prodrugs (**9**); and antibody‒bottlebrush conjugates (ABCs) combining high drug loading, stability, and conditional activation (**10**).

To further enhance controllability, researchers designed a tandem prodrug system (**3**). For instance, the *β*-gal prodrug releases its parent drug when tumor cells overexpress *β*-galactosidase, but *β*-gal expression in macrophages or senescent cells may cause off-target toxicity. Similarly, PA289 is a prodrug payload incorporating a tumor-specific *β*-glucuronide trigger that remains minimally toxic until internalization by target cancer cells and subsequent lysosomal cleavage by *β*-glucuronidase^55^. In parallel, the ADC Isumab01-PL-PA289, generated using PermaLink coupling technology, exhibits exceptional plasma stability^56^. In FRA-positive JEG-3 xenograft models, a single dose of 2.2 mg/kg induces complete tumor regression within 30 days. The Yin team constructed tandem activated gaged galactoside (TACG) (**4**) by introducing a masking group on the galactose hydroxyl^57^. Only under specific stimuli (light, redox, bioorthogonal interactions, etc.) is the galactose hydroxyl unlocked, followed by *β*-galactosidase-mediated drug release, achieving dual activation *via* “AND gate logic”. This strategy can be extended to glucoside, glucuronic acid glucoside, and *N*-acetylglucosamine glucoside prodrugs, providing a universal design approach for carbohydrate drug development.

Monomethyl auristatin E (MMAE) and pyrrolobenzodiazepine (PBD) dimers are ADC payloads widely used in clinical settings. However, their ultra-high potency and unavoidable nonspecific release severely constrain administration at clinically tolerable doses. Li et al.^58^ reported a nitroaromatic linker-based “pro-prodrug” design (**5**) that confines MMAE activation to hypoxic TMEs through nitroreductase-mediated cleavage, while preserving a low-toxicity, poorly permeable prodrug form in normoxic tissues. An analogous strategy using azobenzene linkers (AZO-ADC-2) (**6**) sterically suppresses MMAE activity under normoxia but undergoes azoreductase-triggered cleavage in hypoxic TMEs, resulting in a “switched-on” payload release and a substantially broadened therapeutic window, with *in vivo* MTDs of 200-240 mg/kg^59^. A pro-drug concept (proPBD-ADC) (**7**) was incorporated by shielding the PBD payload with a capping structure that is stable in circulation but conditionally released by tumor-associated proteases or under reducing conditions^60^. Data from rat tolerability studies with HER2-proPBD-ADCs suggest that the trastuzumab ADC incorporating a capped pro-PBD exhibited a higher maximum tolerated dose (MTD, ∼20 mg/kg) compared with the corresponding PBD-based ADC (∼15 mg/kg). In addition, different cap structures of proPBD-ADCs, as well as the use of PBD payloads with attenuated potency, have differential impacts on efficacy and tolerability.

Redox-responsive payloads have likewise been explored to mitigate systemic toxicity. For example, the Cet-C8Pt(IV) ADC (**8**), which couples an anti-EGFR antibody to a stable platinum(IV) prodrug, achieves tumor-localized delivery followed by rapid reduction to the active platinum(II) species in highly reductive microenvironments, thereby inducing tumor-specific DNA damage^61^. Similarly, Lin et al.^62^ introduced a *trans*-cyclooctene (TCO) caging group onto doxorubicin and conjugated the resulting prodrug to an anti-HER2 antibody *via* a pH-sensitive linker, yielding a tumor-enriched prodrug-ADC (TCO-Dox-Ab) (**9**)^62^. The TCO moiety temporarily shields Dox's active amino group. Drug restoration is triggered by an exogenous 3,6-dimethyl-1,2,4,5-tetrazine (Me_2_Tz) initiator *via* an inverse electron-demand Diels‒Alder (iEDDA) reaction, which removes the TCO cage. This strategy integrates bioorthogonal chemistry with TME responsiveness, establishing a dual-control mechanism of “endogenous release and exogenous activation”. This facilitates deeper tumor penetration and supports selective intracellular activation upon administration of a small-molecule trigger.

Recently, an innovative strategy termed antibody-bottlebrush prodrug conjugates (ABCs) (**10**) has been introduced^63^. Bottlebrush prodrugs (BPDs) consist of a central polymer backbone from which flexible side chains emanate, with drug molecules appended to the side chains through cleavable linkers (such as ester bonds) and sterically shielded by PEG “bristles” to maintain a prodrug state. Tetrazine “click” motifs installed at the terminus of the backbone react efficiently with TCO “handles” displayed on the antibody surface under mild physiological conditions, yielding the final ABC constructs. This architecture increases drug loading and systemic stability while conferring antibody-specific conjugation and conditional activation, thereby representing a new frontier in ADC payload delivery design.

Overall, the linker module has evolved beyond a passive bridge that merely tethers antibodies to cytotoxic agents, emerging instead as a central regulator that dictates when and where drug release occurs in ADCs. Through conditional activation, multistep release, or logic-gated designs, linkers can restrict payload activity to the TME and limit collateral damage to healthy tissues. Concurrently, prodrug engineering and triggerable features embedded within the payload itself allow toxicity and pharmacological activity to be tuned with greater precision. As understanding of the TME continues to deepen and advances in smart carriers and bioorthogonal chemistry progress, this modular design paradigm is poised to transform ADCs into precise and controllable therapeutics.

### 2.3 Exogenous activation

Endogenous activation of ADCs relies on TME signals for selectivity, yet limitations persist: tumor heterogeneity and dynamic microenvironment changes can lead to uneven activation levels, while certain signals may also exist in normal tissues, increasing off-target risks. Exogenous activation modalities address these limitations by introducing externally controllable triggers, such as light or radiation, allowing ADC activity to be restricted to defined locations and time points with high precision. RM-1929 (cetuximab sarotalocan), developed by Rakuten Medical, is currently the only clinically approved photosensitizer-based ADC (Fig. 5A). This agent consists of the anti-EGFR antibody cetuximab covalently conjugated to the photosensitizer IR700 and is based on a dual-selectivity concept: antibody-mediated tumor accumulation followed by localized near-infrared (NIR) irradiation to activate the photosensitizer and induce tumor cell death (Fig. 5B)^64^. Clinical studies have demonstrated favorable antitumor efficacy and safety; however, the choice of photosensitizer and the limited tissue penetration of light restrict its broader application^65^.

**Figure 5:**
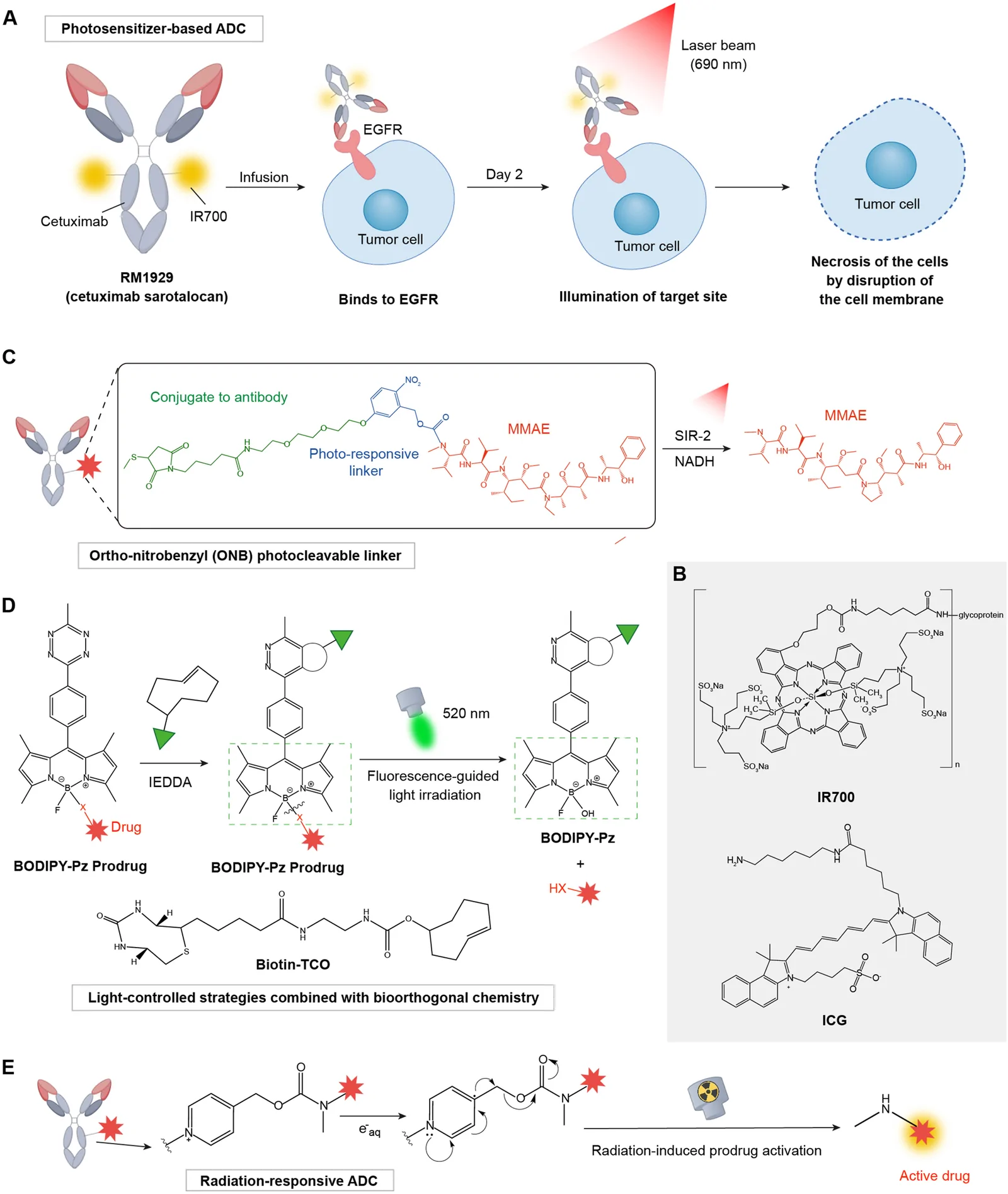
Light- and radiation-activated ADC strategies. (A) RM-1929 (cetuximab sarotalocan), a photosensitizer-based ADC composed of cetuximab conjugated to IR700, enabling antibody-directed tumor targeting followed by near-infrared (NIR) light activation to induce tumor cell death. (B) Chemical structures of IR700 and indocyanine green (ICG). (C) A photocaged ADC incorporating an *ortho*-nitrobenzyl (ONB) linker to mask MMAE; drug release occurs upon silicon-rhodamine-mediated photoreduction under 660 nm irradiation in the presence of NADH. (D) A tetrazine-masked BODIPY–SN-38 prodrug activated *via* bioorthogonal inverse electron-demand Diels‒Alder (iEDDA) reaction and subsequent light-triggered photolysis, enabling image-guided drug release. (E) A radiation-responsive ADC employing an *N*-alkyl-4-picolinium (NAP) linker; radiotherapy-generated hydrated electrons induce linker cleavage and site-specific MMAE release.

To extend the scope of photoactivation, subsequent efforts have employed clinically established dyes such as indocyanine green (ICG) to construct NIR-activatable ADCs. In these systems, ICG is coupled to antibodies through stable linkers, ensuring that payload activation occurs only upon external NIR irradiation (Fig. 5B)^66^. Preclinical studies show that these ADCs remain inert in the absence of light, with no detectable toxicity or antitumor activity *in vitro* or *in vivo*. Upon irradiation, robust local antitumor effects are observed without body weight loss or systemic adverse effects, even when combined with immune checkpoint inhibitors, highlighting the strong spatial control and favorable safety profile of this approach. Further improvements in spatial precision have been achieved through multi-condition, light-gated ADCs. One example employs an *ortho*-nitrobenzyl (ONB) photocleavable linker to conjugate MMAE, rendering the ADC inert under physiological conditions (Fig. 5C)^67^. Payload release requires the simultaneous presence of a silicon-rhodamine photocatalyst, the reducing cofactor NADH, and 660 nm near-infrared irradiation, thereby strictly confining MMAE activation to the illuminated region. Consistent with this design, the ADC remains completely inactive in the absence of light but releases active toxin upon irradiation, resulting in potent cytotoxicity and favorable safety profiles in both cellular systems and tumor-bearing mouse models.

What's more, light-controlled strategies can be combined with bioorthogonal chemistry to construct theranostic prodrug systems with enhanced precision. Tz-BODIPY-SN-38 employs a tetrazine moiety as a dual quencher of fluorescence and cytotoxicity, keeping the drug pharmacologically silent during circulation (Fig. 5D)^68^. Using a pretargeting approach, such as biotin-TCO-mediated tumor localization, subsequent iEDDA reactions remove the tetrazine group, restoring Boron-dipyrromethene (BODIPY) fluorescence. This imaging signal then guides visible-light irradiation, which triggers BODIPY photolysis and releases active SN-38, enabling image-guided, localized drug activation.

Other exogenous energy sources can also activate ADCs. Radiation therapy-generated hydrated electrons (e_aq_^−^) have been exploited as triggers for radiation-responsive ADCs. In one example, an *N*-alkyl-4-picolinium (NAP) masking strategy was used to construct a prodrug ADC, in which MMAE was linked to the anti-FAP antibody sibrotuzumab *via* a NAP carbamate (NAPC, Fig. 5E)^69^. During localized radiotherapy, hydrated electrons selectively reduce the NAP moiety, inducing linker cleavage and releasing active MMAE at the irradiated tumor site. This approach introduces a new physical activation dimension for ADCs by directly coupling drug release to radiotherapy.

Taken together, exogenous activation approaches introduce precise spatiotemporal control into ADC design and establish a versatile design paradigm for safe and effective payload delivery, particularly in the context of tumor heterogeneity and dynamic microenvironments. However, clinical translation remains constrained by practical considerations, including limited optical penetration, dependence on radiation conditions, and device requirements, which may restrict applicability in deep-seated or widely metastatic disease.

## 3 Clinical research cases of typical conditionally active ADCs

Leveraging these diverse design strategies, multiple conditionally active ADCs have advanced to clinical translation (Table 1). Preliminary trial data presented at recent conferences such as AACR and ESMO have provided initial validation of their safety profiles and antitumor potential.

**Table 1:** Clinical research cases of typical conditionally active ADCs.

Drug name	Target	Payload	Development phase	Conditions	Type	Description
CX-2029	CD71	Monomethyl auristatin E (MMAE)	Ⅱ NCT03543813	Head and neck squamous-cell carcinoma, nonsmall-cell lung cancer, esophageal, and diffuse large B-cell lymphoma	Probody™ ADC	• Mask prevents/significantly reduces binding in normal tissue • Proteases cleave substrate, remove mask and allow target engagement in the TME
CX-2009	CD166	DM4	Ⅰ/Ⅱ NCT03149549, NCT04596150	Hormone-receptor-positive/HER2-negative breast cancer and in triple-negative breast cancer		
CX-2051	EpCAM	Camptothecin-derived topoisomerase I inhibitor	I NCT06265688	Advanced solid tumors		
BA3011	AXL	MMAE	Ⅱ NCT04681131	Nonsmall-cell lung cancer	Conditionally active biologic™ ADC	• Introduce pH-responsive structures into the antibody binding domain to inhibit normal tissue binding at neutral pH • Acidic microenvironment triggers protonation to release the inhibition and restore tumor-targeted binding
			Ⅱ NCT04918186	Ovarian cancer		
			Ⅰ/Ⅱ NCT03425279	Solid tumors		
BA3021	ROR-2	MMAE	Ⅱ NCT05271604	PD-1/L1-failure patients with ROR-2 expression in recurrent or metastatic squamous-cell carcinoma of the head and neck		
			Ⅱ NCT04918186	Ovarian cancer		
			Ⅰ/Ⅱ NCT03504488	Nonsmall-cell lung cancer, triple-negative breast cancer, melanoma, and head and neck cancer		
RM-1929	EGFR	IRDye 700DX	I/IIa NCT02422979	Recurrent head and neck squamous cell carcinoma	Photosensitizer-based ADC	• Photoimmunotherapy. • Localized light activation • Disrupt cell membranes, inducing cell death
YL201	B7-H3	Novel topoisomerase I inhibitor	I/Ib NCT05434234, NCT06057922	Advanced solid tumors, extensive-stage small cell lung cancer, nasopharyngeal carcinoma, and pulmonary lymphoepithelial carcinoma	Tumor microenvironment activable linker-payload (TMALIN) platform	• Dual cleavage mechanism. • Cleaved by extracellular proteases in TME while undergoing further cleavage by lysosomal proteases within cells
YL202	HER3		I NCT05653752	Heavily pretreated non-small cell lung cancer and breast cancer		
ZL-1310	DLL3		I NCT06179069	Extensive-stage small cell lung cancer		

### 3.1 Probody platform

The Probody platform, developed by CytomX Therapeutics (WO20100968), is a representative example of protease-triggered masking antibody technology. It fuses a masking module at the N-terminus of the antibody light chain, which includes a blocking peptide, uPA substrate peptide, and flexible linker^18^. This module blocks antigen binding in normal tissues, reducing affinity by 40-fold. In the TME, uPA specifically cleaves the substrate, removing the mask and restoring antibody activity. Several ADC therapies utilizing this technology are currently in clinical trials.

CX-2029. CD71 (transferrin receptor 1), highly expressed on tumor cells for iron uptake and proliferation, is also widespread in normal tissues like erythroid precursors, limiting the therapeutic window of conventional ADCs. To address this, the conditionally active ADC CX-2029 was developed using the Probody technology platform^70^. This drug incorporates a propeptide masking domain (47 amino acids) at the N-terminus of the anti-CD71 antibody light chain, which is specifically cleaved by tumor-associated proteases, ensuring activation only within the TME. It utilizes a *vc*-MMAE linker with a drug-to-antibody ratio (DAR) ∼2. A Phase I clinical study established 3 mg/kg as the recommended Phase II dose (NCT03543813), demonstrating an acceptable safety profile and observed partial responses in patients with squamous cell carcinoma^11^. Dose-limiting toxicities were anemia and neutropenia, likely due to CD71 on erythroid precursors and myelosuppressive effects of MMAE. This prodrug approach restricts active drug release to tumors, widening the therapeutic window despite residual target-associated toxicity. Furthermore, pharmacokinetics showed CX-2029 circulates mainly as an intact prodrug (>90%), with minimal free MMAE (≤4.3%), indicating low systemic exposure^70^.

CX-2009. Similarly, CX-2009 targets the traditionally “undruggable” CD166. It is conjugated to DM4 *via* a cleavable linker, with its antibody masked by a peptide attached through a protease-cleavable linker (DAR ∼3.5). In Phase I/II trials, 7 mg/kg every three weeks was established as the recommended dose, demonstrating good tolerability with no dose-limiting toxicities (NCT03149549, NCT04596150)^71^. Toxicities were consistent with the DM4 payload, including ocular toxicity (49%, primarily keratitis), liver function abnormalities (aspartate aminotransferase elevation 8%, alanine aminotransferase elevation 5%), and anemia (5%); most events were Grade 1–2 and manageable. Pharmacokinetic data showed >90% of CX-2009 circulated as intact prodrug. While both ADCs use a prodrug strategy for widely expressed targets, their distinct efficacy and safety profiles are jointly determined by the specific target biology and payload.

CX-2051. CX-2051 targets EpCAM and has demonstrated significant potential in Phase I trials^22^. The antibody's masking activity can be blocked by masking peptides, with masking efficiency exceeding 100-fold in cell binding assays^72^. It is activated exclusively in the TME by specific proteases, releasing a novel topoisomerase-1 inhibitor (camptothecin analog) and enhancing the bystander effect through an optimized linker. Among 18 evaluable patients with advanced colorectal cancer, the objective response rate (ORR) was 28% (5/18), with the highest dose group (10 mg/kg, Q3W) achieving an ORR of 43% (3/7)—significantly outperforming existing third-line and later treatments (typically single-digit ORRs). The disease control rate (DCR) was 94% (17/18), with a median progression-free survival (PFS) of 5.8 months (NCT06265688).

### 3.2 Conditionally active biologics (CAB) technology platform

BA3011. AXL, a receptor tyrosine kinase overexpressed in various solid tumors, is linked to poor prognosis through its roles in progression, metastasis, and therapy resistance^73^. The ADC BA3011 targets AXL using a pH-dependent CAB platform^74^. It conjugates a humanized IgG1 to MMAE *via* a cleavable linker (DAR ∼4) and employs PaCS technology to bind with high affinity in the acidic tumor microenvironment while reducing binding at physiological pH. *In vitro*, it showed potent activity at pH 6.0 (EC_50_ = 4.78 ng/mL). Population PK analysis indicates a two-compartment model with linear and nonlinear elimination: BA3011 clearance was 1.47 L/day and released MMAE clearance was 3.85 L/day^75^. In clinical trials (NCT04681131), monotherapy achieved an ORR of 27.8% and DCR of 55.6%, with responses even in low-AXL-expressing patients (median duration of response [DoR] 4.8 months)^39^. Most adverse events were manageable Grade 1‒2, with drug exposure-linked toxicities such as neutropenia.

BA3021. BA3021 is a pH-dependent ADC targeting ROR2 (pH inflection point = 6.4), which exhibits high affinity and efficient internalization under acidic conditions^76^. In a Phase II trial involving heavily pretreated, PD-1 inhibitor-resistant head and neck squamous cell carcinoma patients (NCT05271604, *n* = 31, 1.8 mg/kg dose), the evaluable population (*n* = 28) achieved a 36% ORR (1 complete, 9 partial responses) and an 86% disease control rate (DCR)^38^. Median DoR was not reached (>3.6 months), indicating durable efficacy. Adverse events were primarily Grade 1‒2 (*e.g.*, fatigue 59%, anemia 34%, nausea 34%). Only 6 and 2 patients experienced Grade 3 and 4 events (hyponatremia and neuropathy), with no Grade 5 events, confirming overall good tolerability.

### 3.3 RM-1929

In an open-label Phase I/IIa clinical trial (NCT02422979) for locally recurrent head and neck squamous cell carcinoma (rHNSCC), investigators evaluated the clinical value of RM-1929 (cetuximab sarotalocan) photoimmunotherapy^65^. This drug is formed by covalently conjugating cetuximab, which targets EGFR, with the photoactivated dye IR700, introducing “local light activation” as a key condition. The study employed a two-phase design: first, dose escalation to determine the recommended dose (640 mg/m^2^), followed by an assessment of safety and preliminary efficacy in refractory patients combined with local tumor irradiation (690 nm non-thermal red light). Results demonstrated good tolerability with no dose-limiting toxicities, and the majority of adverse events were mild to moderate. The regimen achieved an unconfirmed ORR of 43.3% and a confirmed ORR of 26.7% in patients with multi-line-treated rHNSCC, with a median overall survival of 9.3 months.

However, RM-1929 photodynamic immunotherapy faces key challenges: its photoactivation relies on localized light delivery, limiting its use to superficial or accessible lesions, and its complex treatment regimen reduces patient compliance and adds operational risk. Future strategies aim to address these by focusing on technology innovation: developing deeper-penetrating NIR-II photosensitizers and minimally invasive/wearable light systems; and on antibody engineering, such as exploring bispecific designs and optimized conjugation chemistry to improve targeting, efficacy, and safety.

### 3.4 Tumor microenvironment activable linker-payload (TMALIN) platform

The TMALIN platform is a conditionally activated ADC technology developed by MediLink Therapeutics. It employs a valyl dipropyl-lysyl glycinamide-methylene (-VK∗G-NHCH_2_-O-) enzyme-cleavable sequence combined with irreversible pyrimidine coupling anchor for spatial conformation protection, ensuring high systemic stability (*in vitro* payload drop-off <2% over 21 days)^10^^,^^40^. Within tumor tissue, the platform enables dual-specificity release. Intracellularly, the ADC is cleaved by cathepsin B in lysosomes to kill antigen-positive cells. Extracellularly, soluble proteases (*e.g*., MMPs) in the TME cleave the linker, releasing a membrane-permeable payload that kills adjacent antigen-low or antigen-negative tumor cells *via* a bystander effect, effectively overcoming tumor heterogeneity. Several TMALIN-based candidates are now in clinical studies, showing promising results.

*YL201*. YL201 is a B7-H3-targeting ADC that conjugates a humanized antibody to a novel topoisomerase I inhibitor *via* a protease-cleavable linker, forming a stable circulating prodrug. Its linker is sequentially cleaved by extracellular tumor proteases and then intracellular lysosomes for precise payload release. In a Phase I/Ib trial of heavily pretreated advanced solid tumors (NCT05434234, NCT06057922), YL201 showed broad antitumor activity^77^. ORRs were 63.9% in extensive-stage small cell lung cancer (median PFS 6.3 months), 48.6% in nasopharyngeal carcinoma (median PFS 7.8 months), and 54.2% in pulmonary lymphoepithelioma-like carcinoma (median PFS 8.1 months). Despite this efficacy, key non-target toxicities were rare: treatment-related interstitial lung disease and infusion reactions occurred in only 1.3% and 0.3% of patients, respectively. Just 5.4% of patients discontinued treatment due to toxicity. Phase 3 clinical trials for SCLC and NPC have already been initiated (NCT06612151 and NCT06629597). YL201 has received U.S. FDA Breakthrough Therapy Designation, making it the first B7-H3 ADC to achieve this status.

*YL202*. Another candidate drug, YL202 (targeting HER3), employs an anti-HER3 IgG1 monoclonal antibody linked *via* a tripeptide spacer to the novel topoisomerase I inhibitor YLO010014, achieving a high drug‒antibody ratio (DAR 8). In a Phase I trial in heavily pretreated non-small cell lung cancer and breast cancer patients (NCT05653752), it showed promising efficacy at therapeutic doses, with an ORR of 41.0% and a DCR of 94.9% across tumor types^78^. In breast cancer patients specifically, the ORR was 54.5% and the DCR was 100%. However, the drug's development faced a safety challenge. In 2024, the FDA placed a partial clinical hold on the U.S. trial, which was lifted two months later following data review and protocol amendments, limiting the dose to no more than 3 mg/kg.

Additionally, DLL3 ADC (ZL-1310) of Zai Lab demonstrated an ORR of 67% in its Phase I trial (NCT06179069) among 74 evaluable small cell lung cancer patients receiving second-line treatment^79^. Notably, the ORR reached 79% at the 1.6 mg/kg dose. Multiple other compounds, including YL242, YL211, and XNW27011, are in preclinical research phases, demonstrating significant potential for clinical application^80^, ^81^, ^82^.

## 4 Conclusions and perspectives

Conditionally active ADCs are redefining targeted chemotherapy by integrating antibody specificity with TME cues and, in some designs, external triggers. This approach allows payloads to remain inert in circulation and activate selectively within tumors, enhancing efficacy while minimizing systemic toxicity. Clinical and preclinical studies of platforms such as CX-2029 (Probody) and BA3011 demonstrate that endogenous conditional activation can localize drug release, reduce free circulating payload, and expand the therapeutic window^11^^,^^74^. More broadly, these strategies exemplify a new design paradigm: ADCs are no longer passive drug carriers but logic-gated systems capable of precise spatial and temporal control. Nevertheless, several challenges remain in the clinical and translational development of conditionally active ADCs.

### 4.1 TME heterogeneity and predictability of activation

The spatial and temporal heterogeneity of the TME is a key factor contributing to variability in clinical responses. Differences in protease activity, pH, redox state, and other metabolic features exist across tumor subtypes and within distinct regions of the same tumor. Additionally, some activating signals are present in normal tissues or inflamed sites, increasing the risk of off-target payload release. Clinical observations, such as mild to moderate ocular and hepatic toxicities in CX-2009 treatment, highlight that single-input endogenous triggers cannot fully ensure universality and precision^71^. Strategies to address these challenges include multi-input, logic-gated ADC designs that integrate endogenous cues with exogenous controls to improve activation specificity, complemented by high-resolution imaging and biomarker monitoring for real-time feedback and adaptive dosing.

### 4.2 Payload release kinetics and linker design

Balancing stability and cleavability of the linker remains a core challenge. Overly labile linkers compromise safety, whereas excessively stable linkers can hinder effective payload release and reduce efficacy. Next-generation conditionally active ADCs employ prodrug payloads, enzyme-specific or redox-responsive linkers, combined with fluorescent or imaging reporters, to achieve controlled release with concurrent visualization. For instance, CAT-Fluor ADCs utilize Cathepsin B-cleavable linkers to simultaneously release cytotoxic payloads and unquench fluorescent signals, enabling subcellular-level, *in situ* monitoring^83^. Such designs facilitate pharmacokinetic optimization and provide direct data to support clinical efficacy evaluation and individualized therapy adjustments. The continued development of intelligent linker-prodrug systems will further enhance precision and predictability in payload delivery.

### 4.3 Exogenous trigger strategies and deep-tumor activation

Light-activated ADCs and photoactivatable antibody‒nucleic acid conjugates illustrate the potential of combining external physical stimuli (near-infrared or ultraviolet light) with endogenous biochemical conditions to achieve dual-gated, spatiotemporally precise payload release^84^. However, limited tissue penetration of UV and near-infrared light constrains clinical applicability. Future directions include the development of high-penetration photosensitizers, minimally invasive illumination devices, or alternative physical triggers (ultrasound, magnetic fields, thermosensitive systems) to expand controllable activation in deep and multifocal tumors. Integrating multiple activation modalities with TME cues may further enhance precision in complex tumor contexts.

### 4.4 Intelligent, programmable design and combination therapy potential

Logic-gated design frameworks provide a blueprint for “smart” drug release, enabling fine-tuned control of dosage and timing, while also optimizing synergy with immunotherapies or TME modulators. Localized cytotoxic payload release can induce immunogenic cell death, reducing systemic immunosuppression and providing a mechanistic basis for rational combination with immune checkpoint inhibitors. Computational modeling and AI are anticipated to play pivotal roles in optimizing activation thresholds, predicting release kinetics, and stratifying patients, ultimately advancing programmable, visualizable, and patient-tailored therapeutic platforms.

### 4.5 Clinical translation and sustainable development

Despite the promise of conditionally active ADCs, large-scale clinical translation faces challenges in manufacturing complexity, batch uniformity, and cost. The integration of site-specific conjugation, intelligent prodrug chemistry, and multi-modal activation strategies represents a critical technological pathway. Coupled with the assessment of endogenous TME heterogeneity and optimization of exogenous activation, these advances are expected to enhance clinical feasibility and predictability, establishing a foundation for personalized oncology.

In summary, conditionally active ADCs move beyond conventional antigen-targeted delivery toward smart, logic-gated, and spatiotemporally precise therapeutic systems. By combining intelligent design, multi-modal activation, and real-time monitoring, they offer a platform capable of improved safety, enhanced efficacy, and personalized treatment, paving the way for the next generation of targeted cancer therapies.

## Author contributions

Yuxi Wang put forward valuable suggestions regarding topic selection, conceptual framework and manuscript revision; Cuiyu Guo drafted the manuscript and drew pictures and tables; Yaqin He compiled the materials and drafted the initial draft; Ying Lu and Qilin Dun revised the manuscript; Jinliang Yang offered supplementary guidance and revised the manuscript. All authors reviewed and approved the final version submitted for publication.

## Conflicts of interest

The authors declare no competing financial interest.
